# Percutaneous Large Bore Aspiration Thrombectomy of Tumor Embolism Causing Massive Pulmonary Embolism

**DOI:** 10.1016/j.jaccas.2022.01.008

**Published:** 2022-03-16

**Authors:** Antony Gayed, Stephen Stringfellow, Ricardo Yamada, Thor Johnson, Chelsea Wright-Void, Christopher Hannegan

**Affiliations:** Division of Vascular and Interventional Radiology, Department of Radiology, Medical University of South Carolina College of Medicine, Charleston, South Carolina, USA

**Keywords:** cancer, chest pain, postoperative, pulmonary circulation, right ventricle, thrombus, CT, computed tomography, ICU, intensive care unit, IVC, inferior vena cava, LPA, left pulmonary artery, PA, pulmonary artery, RCC, renal cell carcinoma, TEE, transesophageal echocardiogram

## Abstract

We used aspiration thrombectomy to treat a 66-year-old man with renal cell carcinoma undergoing radical nephrectomy and caval thrombectomy with a massive pulmonary artery tumor embolism. (**Level of Difficulty: Intermediate.**)

## Central Illustration

**Figure:**
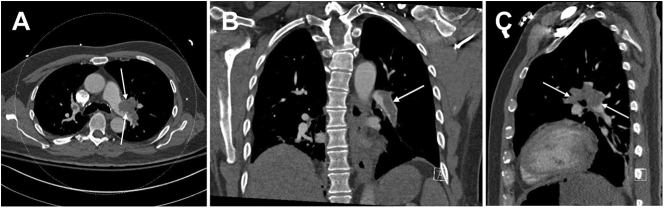

## History of Presentation

A 66-year-old man presented with hematuria, anemia, and weight loss and was found to have an 11-cm right renal neoplasm with tumor expanding the renal vein and a nonobstructive thrombus extending 4.7 cm into the infrahepatic inferior vena cava (IVC). There was no evidence of metastatic disease on staging computed tomographies (CTs) or bone scintigraphy. His chronic comorbidities were controlled with medication, and his Eastern Cooperative Oncology Group performance status was zero. He elected for single-stage open right nephrectomy and IVC tumor thrombectomy with urology and transplant surgery. Intraoperative monitoring included radial artery catheter blood pressure monitoring and transesophageal echocardiogram (TEE). Operative inspection revealed large right renal cell carcinoma (RCC) expanding the renal vein. After exposure of the right kidney, but before control of the suprarenal IVC was gained, decreased caliber of the tumor-involved renal vein was noted. TEE revealed a new right heart thrombus visible for several cardiac cycles before passing into the pulmonary circulation. The patient’s condition became hemodynamically unstable, with blood pressure 68/49 mm Hg and heart rate 111 beats/min, requiring bolused isotonic intravenous fluids, 1 U packed red blood cells, and continuous administration of 3 vasopressors. Cardiothoracic surgery provided intraoperative consultation and advised against attempting immediate open thrombectomy. Given the patient’s moribund status and with single-stage margin-negative resection no longer possible, IVC tumor resection was foregone, and nephrectomy was expedited by stapling across the tumor-involved renal vein–IVC confluence. The patient was admitted to the intensive care unit (ICU) on norepinephrine 16 μg/min, epinephrine 0.1 μg/kg/min, and vasopressin 0.04 U/min, which were weaned over the ensuing 24 hours, and the patient was extubated in the ICU. Interventional radiology was consulted to discuss further management of the pulmonary embolism (PE) and IVC thrombus. Physical examination showed a heart rate of 114 beats/min, blood pressure of 141/71 mm Hg, SpO_2_ 90% to 92% on 2 L nasal cannula, respiratory rate 23 breaths/min. The patient was awake and experiencing mild to moderate distress Neurologically the patient was alert and oriented and showed no focal deficits. Cardiovascular examination showed tachycardia and well-perfused extremities. Pulmonary signs included increased work of breathing. Abdominal examination showed tenderness along the surgical incision and a clean surgical dressing. Skin examination showed a right internal jugular catheter. On musculoskeletal inspection the patient had symmetrical bulk.

**Learning Objectives**

• To recognize that renal cell carcinoma tumor embolism is a complication of venous invasive disease resection with very high mortality.
• To understand the role of large bore aspiration thrombectomy as an adjunct to traditional surgical management of tumor embolism.

## Medical History

The patient’s medical history revealed hypertension controlled with amlodipine and lisinopril, hyperlipidemia for which the patient was taking atorvastatin, and benign prostatic hyperplasia for which the patient was taking tamsulosin. The patient described using 1 alcoholic beverage per week and did not describe smoking or recreational drug use. His family history included unspecified malignancies in his father and paternal grandmother and hypertension in his mother. He had previously undergone surgery for inguinal hernia repair.

## Differential Diagnosis

The differential diagnosis included: 1) massive tumor pulmonary embolism; 2) massive bland pulmonary embolism; and 3) hypotension secondary to acute blood loss.

## Investigations

**Figure 1:**
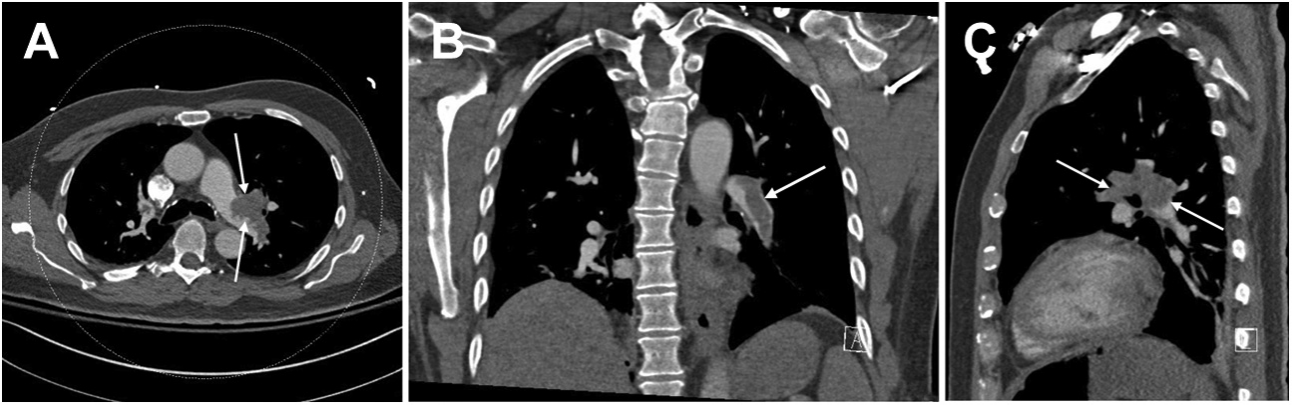
Computed Tomography Pulmonary Angiography of Central Pulmonary Embolism Computed tomography pulmonary angiographic view demonstrating near-occlusive left pulmonary artery thrombus in **(A)** axial, **(B)** coronal, and **(C)** sagittal multiplanar reformats **(arrows)**.

**Figure 2:**
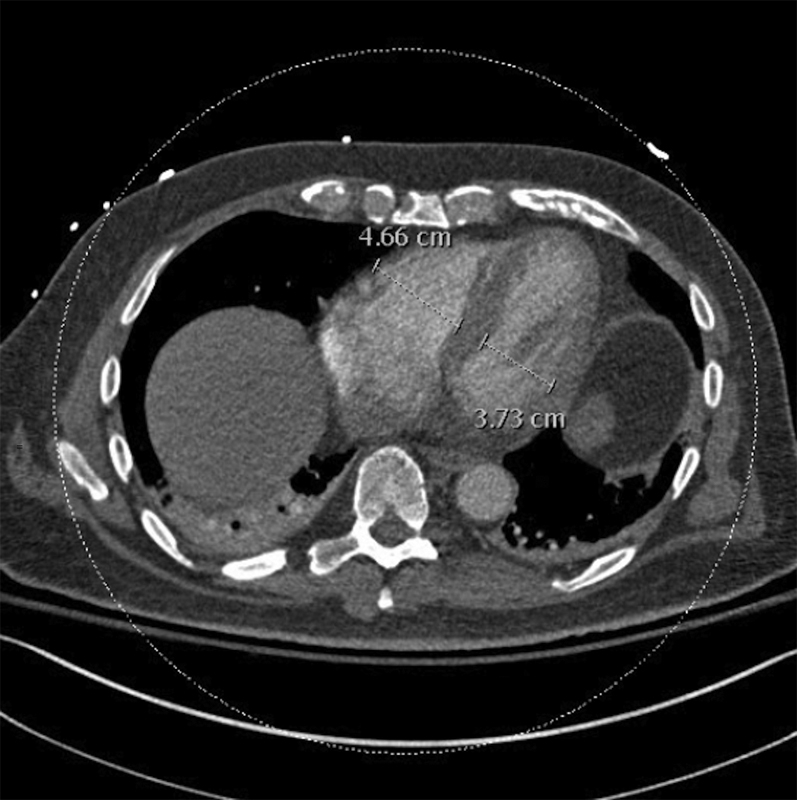
Computed Tomography Pulmonary Angiography Findings of Right Heart Strain Abnormal right ventricle: left ventricle ratio of 1.2.

**Figure 3:**
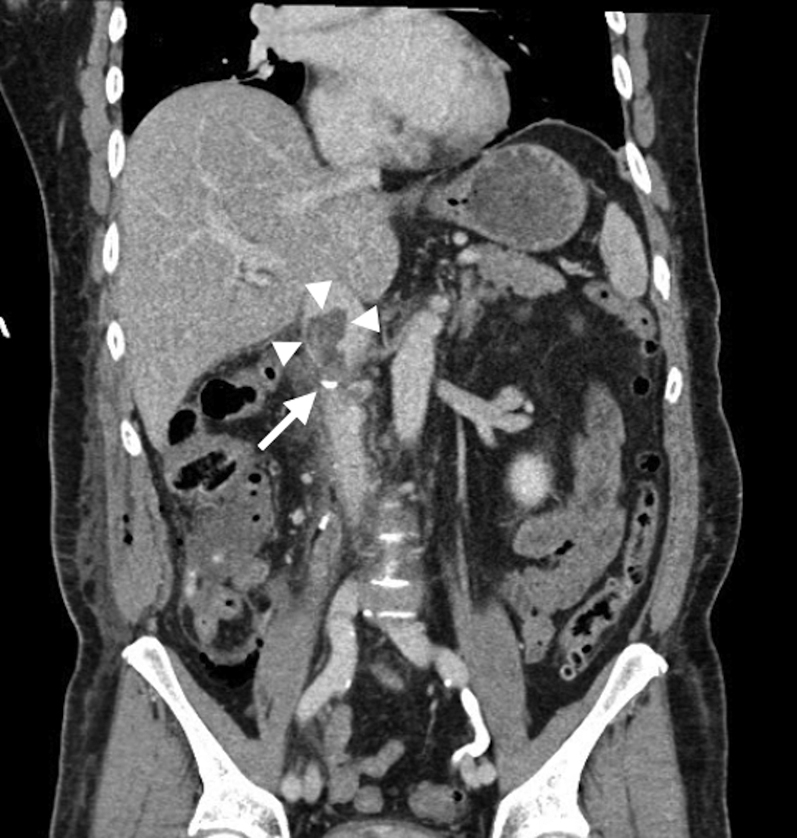
Computed Tomography of Inferior Vena Cava Tumor Thrombus Coronal computed tomography demonstrating inferior vena cava thrombus **(arrowheads)** adjacent to a nephrectomy staple **(arrow)**.

The postoperative laboratory results were notable for hemoglobin of 7.7 U compared with the preoperative value of 10.0 U. CT pulmonary angiography demonstrated a nearly occlusive thrombus in the left pulmonary artery (LPA) (Figure 1). Radiology indicated that it most likely represented tumor embolism, with additional bilateral segmental and distal subsegmental emboli. Right ventricular dilation was also noted, with a right ventricular to left ventricular ratio of 1.2 (Figure 2). Contrast-enhanced abdominopelvic CT demonstrated a 3.9-cm nonocclusive IVC mixed bland and tumor thrombus adjacent to the surgical staple line (Figure 3). No lower extremity thrombus was seen on ultrasound.

## Management

**Figure 4:**
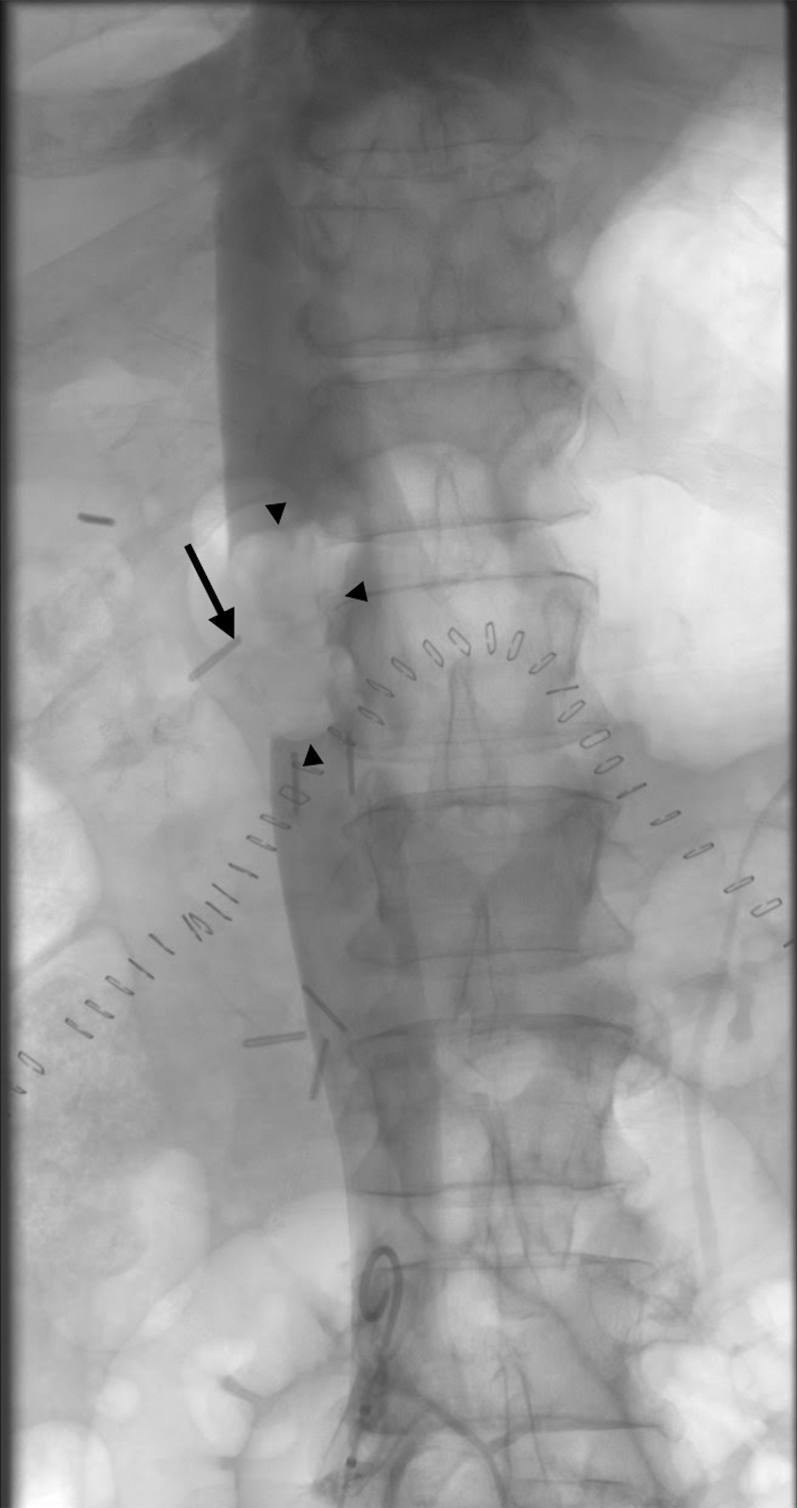
Inferior Venacavagram Inferior venacavagram demonstrating large nonocclusive thrombus **(arrowheads)** extending from the ligated right renal vein **(arrow)**.

**Figure 5:**
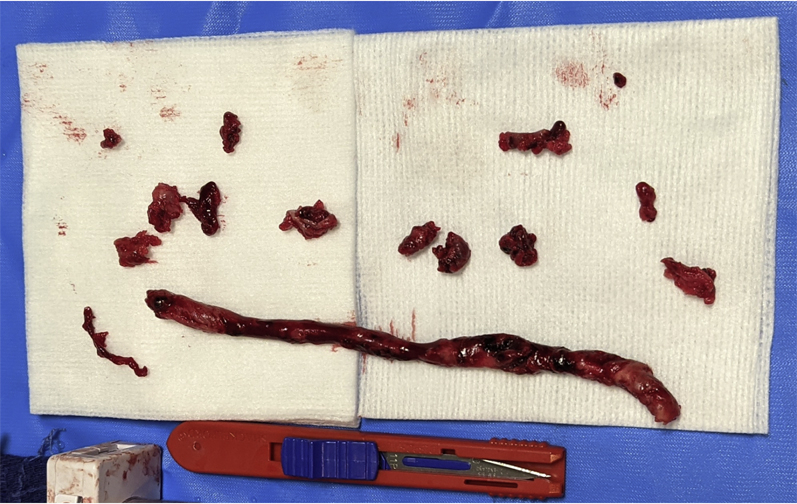
Pulmonary Thrombectomy Gross Pathology Tumor aspirated from the main and left pulmonary arteries. Pathologic examination revealed thrombus with extensive clear cell renal cell carcinoma. (11-cm scalpel for scale.)

**Figure 6:**
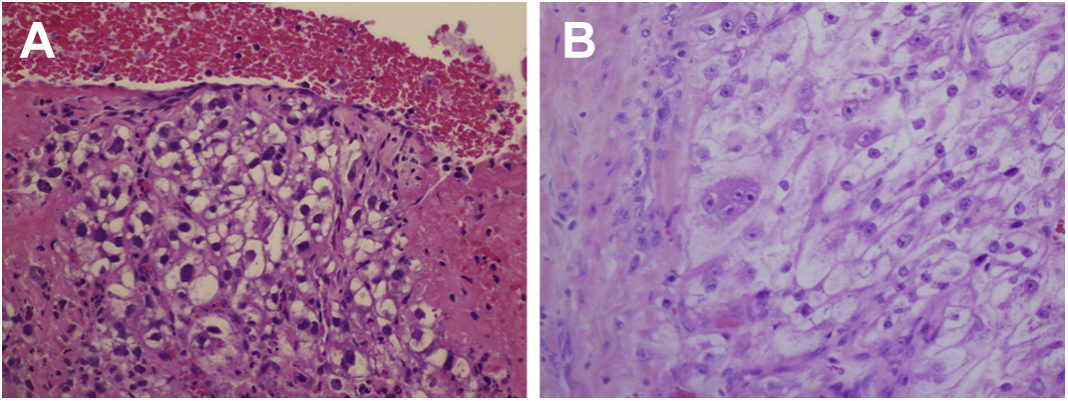
Histopathology Clear cell renal cell carcinoma in intravascular thrombus **(A)** with histologic appearance similar to that of original renal cell carcinoma **(B)**. (Hematoxylin & eosin staining, ×200.)

**Figure 7:**
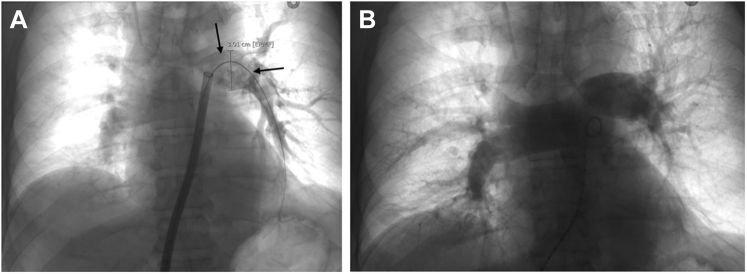
Pulmonary Angiography Pulmonary angiograms before **(A)** and after **(B)** thrombectomy demonstrating near-occlusive left pulmonary artery (LPA) filling defect **(arrows)**, which resolved with thrombectomy. (Left pulmonary artery caliber 3.0 cm.)

With consideration of the patient’s high risk for further decompensation, given the intraoperative hemodynamic collapse, large central pulmonary TE, evidence of right heart strain, risk of further caval thrombus embolization, and contraindication to thrombolysis, endovascular thrombectomy was considered. Upon discussion with the critical care surgeon, transplant surgeon, urologist, and interventional radiologist, the decision was made to attempt endovascular caval and PA thrombectomy. Given the size of the thrombus, a large bore aspiration catheter was required. On postoperative day 1, large bore mechanical aspiration thrombectomy of the IVC thrombus and TE was performed with right common femoral vein access using the Inari FlowTriever mechanical thrombectomy device with the Triever24 and Triever20 curve large bore catheter systems (Inari Medical). The IVC thrombus was densely adherent to the IVC surgical margin aside from small fragments removed, signifying stability of the thrombus (Figure 4). Aspiration thrombectomy of the main PA and LPA successfully extracted a 15-cm tumor thrombus (Figure 5), confirmed with pathological examination (Figure 6). Pulmonary angiography demonstrated resolution of the proximal TE (Figure 7). After thrombectomy, the PA pressure decreased from 40/18 mm Hg (mean 27 mm Hg) to 30/7 mm Hg (mean 16 mm Hg). One day after thrombectomy, a heparin drip was started, a transthoracic echocardiogram showed normal heart size and estimated right atrial pressure, and the patient was transferred from the ICU to the inpatient department.

## Discussion

TE is rare in patients with RCC. Most reported cases of TE occur during concomitant resection of the primary tumor and of caval tumor thrombus extension.^1^ TE occurs in 1.5% of patients with RCC caval tumor thrombus, and the mortality is 75%.^2^ A retrospective study comparing complete caval thrombus removal with incomplete removal demonstrated 5-year survival of 68% in the complete resection patient group vs 17.5% in the incomplete resection group. In a series of 9 patients who underwent planned surgical PA tumor embolectomy at the time of nephrectomy, survival was comparable with that in patients with the same RCC stage without TE. This suggests that in terms of staging, RCC PA TE should be considered an extension of tumor in the vein rather than a distant metastatic disease. In addition to the life-saving physiological benefit of tumor embolectomy in massive and submassive pulmonary embolism, there may also be an oncologic survival benefit.^1^ Whereas there are multiple reports of successful surgical embolectomy after intraoperative TE, survival is considered rare.^2^, ^3^, ^4^ Intraoperative TEE should be considered in all patients with level II to level IV thrombus, thrombus at least 2 cm superior to the involved renal vein.^5^ In this patient’s case, intracardiac and PA thrombus was detected with TEE, at which time the patient became hypotensive. We know of 1 other reported case of catheter thrombectomy of tumor thrombus using 8-F continuous suction, also in the setting of an RCC tumor thrombus, causing massive PE. In that case, the patient was ineligible for surgical embolectomy because of coagulopathy and the risk of hemodynamic collapse with general anesthesia, nor was he eligible for thrombolysis because of retroperitoneal bleeding.^6^ In the present case, the kidney was resected, but caval thrombus resection was not attempted in the operating room because of the patient’s hemodynamic instability and the risk for further tumor embolization. Thrombolysis was contraindicated in the postoperative setting, and would be less effective for tumor thrombus.^6^ Surgical embolectomy is the primary treatment option, but it carries the risk of hemodynamic collapse with induction of anesthesia. This case is unique in that a large bore, 24-F aspiration catheter was required for extraction. This newer tool provides an essential advantage in the removal of large TE and has the capability to remove TE en bloc, as demonstrated in Figure 5. Percutaneous aspiration thrombectomy was successful in this case.

The case study has limitations. First, inasmuch as this patient’s condition had become partially hemodynamically stabile after the intraoperative embolic event, it is harder to associate the technically successful outcome with immediate survival. It is noted that he remained tachycardic, hypoxic, and tachypneic with pulmonary hypertension before the case, with immediate improvement after the procedure, and transfer from the ICU to the inpatient department <1 day after the procedure. Considering the nature of the embolism, a favorable response to lytic therapy and anticoagulation was less likely. Surgical embolectomy would have been more risky, as discussed earlier. Second, the long-term prognosis for this patient remains guarded because of residual tumor, given the likelihood of tumor emboli having showered into small pulmonary arteries and arterioles. However, it is unlikely that total tumor clearance was achievable by any intervention.

## Follow-Up

The patient returned to baseline functional status without shortness of breath and was discharged on postoperative day 7 on oral anticoagulation. He started adjuvant immunotherapy and remained asymptomatic during outpatient follow-up.

## Conclusions

This report adds to the literature an option for TE extraction that can be used in cases of hemodynamic instability without the additive hemodynamic risk of anesthesia induction or cardiopulmonary bypass, and it can be applied in clinical scenarios in which thrombolysis is contraindicated. Additionally, this treatment could reduce perioperative cardiac risk in TE cases, improving patient candidacy for surgical resection or embolectomy.

## Funding Support and Author Disclosures

The authors have reported that they have no relationships relevant to the contents of this paper to disclose.

## References

[1] Kayalar N., Leibovich B.C., Orszulak T.A., Schaff H.V., Sundt T.M. (2010). Concomitant surgery for renal neoplasm with pulmonary tumor embolism. J Thorac Cardiovasc Surg.

[2] Shuch B., Larochelle J.C., Onyia T., Vallera C., Margulis D., Pantuck A.J. (2009). Intraoperative thrombus embolization during nephrectomy and tumor thrombectomy: critical analysis of the University of California-Los Angeles experience. J Urol.

[3] Wang K.R. (2014). Massive postoperative pulmonary artery tumor embolism from renal cell carcinoma. Anesthesiology.

[4] Kobayashi T., Ogura K., Nishizawa K., Muranaka H., Ono H., Oda T. (2004). Successful recovery from a massive pulmonary artery tumor embolism occurring during surgery for renal cell carcinoma. Int J Urol.

[5] Kostibas M.P., Arora V., Gorin M.A., Ball M.W., Pierorazio P.M., Allaf M.E. (2017). Defining the role of intraoperative transesophageal echocardiography during radical nephrectomy with inferior vena cava tumor thrombectomy for renal cell carcinoma. Urology.

[6] Pallister Z.S., Montero-Baker M., Mills J.L., Chung J. (2018). Percutaneous suction thrombectomy of large tumor thrombus causing massive pulmonary embolism. J Vasc Surg Cases Innov Tech.

